# Metallopeptidades 2 and 9 genes epigenetically modulate equine endometrial fibrosis

**DOI:** 10.3389/fvets.2022.970003

**Published:** 2022-08-12

**Authors:** Joana Alpoim-Moreira, Carina Fernandes, Jorge Pimenta, Miguel Bliebernicht, Maria Rosa Rebordão, Pedro Castelo-Branco, Anna Szóstek-Mioduchowska, Dariusz J. Skarzynski, Graça Ferreira-Dias

**Affiliations:** ^1^CIISA - Center for Interdisciplinary Research in Animal Health, Faculty of Veterinary Medicine, University of Lisbon, Lisbon, Portugal; ^2^Associate Laboratory for Animal and Veterinary Sciences (AL4AnimalS), Lisbon, Portugal; ^3^Unidade Estratégica de Investigação e Serviços de Biotecnologia e Recursos Genéticos (UEISBR), Instituto Nacional de Investigação Agrária e Veterinária, I. P. (INIAV), Vairão, Portugal; ^4^Embriovet, Muge, Portugal; ^5^Polytechnic of Coimbra, Coimbra Agriculture School, Coimbra, Portugal; ^6^Faculty of Medicine and Biomedical Sciences (FMCB), University of Algarve, Faro, Portugal; ^7^Algarve Biomedical Center Research Institute (ABC-RI), Faro, Portugal; ^8^Institute of Animal Reproduction and Food Research PAS, Olsztyn, Poland

**Keywords:** endometrial fibrosis, mare, collagen, epigenetics, DNMT, MMP, endometrosis

## Abstract

Endometrium type I (COL1) and III (COL3) collagen accumulation, periglandular fibrosis and mare infertility characterize endometrosis. Metalloproteinase-2 (MMP-2), MMP-9 and tissue inhibitors of metalloproteinases (TIMP-1 and TIMP-2) are involved in collagen turnover. Since epigenetic changes may control fibroproliferative diseases, we hypothesized that epigenetic mechanisms could modulate equine endometrosis. Epigenetic changes can be reversed and therefore extremely promising for therapeutic use. Methylation pattern analysis of a particular gene zone is used to detect epigenetic changes. DNA methylation commonly mediates gene repression. Thus, this study aimed to evaluate if the transcription of some genes involved in equine endometrosis was altered with endometrial fibrosis, and if the observed changes were epigenetically modulated, through DNA methylation analysis. Endometrial biopsies collected from cyclic mares were histologically classified (Kenney and Doig category I, *n* = 6; category IIA, *n* = 6; category IIB, *n* = 6 and category III, *n* = 6). Transcription of *COL1A1, COL1A2, COL3A1, MMP2, MMP9, TIMP1*, and *TIMP2* genes and DNA methylation pattern by pyrosequencing of *COL1A1, MMP2, MMP9, TIMP1* genes were evaluated. Both *MMP2* and *MMP9* transcripts decreased with fibrosis, when compared with healthy endometrium (category I) (*P* < 0.05). *TIMP1* transcripts were higher in category III, when compared to category I endometrium (*P* < 0.05). No differences were found for *COL1A1, COL1A2, COL3A1* and *TIMP2* transcripts between endometrial categories. There were higher methylation levels of (i) *COL1A1* in category IIB (*P* < 0.05) and III (*P* < 0.01), when compared to category I; (ii) *MMP2* in category III, when compared to category I (*P* < 0.001) and IIA (*P* < 0.05); and (iii) *MMP9* in category III, when compared to category I and IIA (*P* < 0.05). No differences in *TIMP1* methylation levels were observed between endometrial categories. The hypermethylation of *MMP2* and *MMP9*, but not of *COL1A1* genes, occurred simultaneously with a decrease in their mRNA levels, with endometrial fibrosis, suggesting that this hypermethylation is responsible for repressing their transcription. Our results show that endometrosis is epigenetically modulated by anti-fibrotic genes (*MMP2* and *MMP9*) inhibition, rather than fibrotic genes activation and therefore, might be promising targets for therapeutic use.

## Introduction

Equine endometrosis is a multifactorial disease considered to be one of the most important causes of equine infertility, especially in older mares (1) and has an economic impact on the horse breeding history (2). Endometrosis is characterized by periglandular fibrosis of the equine endometrium (3) which compromises the integrity and function of the endometrial glands required for embryonic preimplantation and placental development (4). However, this condition is still a puzzle regarding its pathogenesis and treatment. Periglandular arrangement of myofibroblasts, associated with the deposition of extracellular matrix (ECM), such as collagen (COL), is a cardinal feature of endometrosis in mares (5). Bochsler and Slauson (6) stated that the deposition of collagen types I (COL1) and III (COL3) occurs in fibrotic processes, which promotes the development of cicatricial tissues with major tensile strength. In a healthy endometrium, the first collagen to be synthesized is COL3, which in turn is gradually replaced by COL1 following the development of fibrotic lesions (7, 8). Collagen type I is usually predominant in Kenney's category III endometrium (severe fibrosis) (7), and COL3 in category I healthy endometrium (9). Matrix metalloproteinases (MMPs) are a family of extracellular endopeptidases (10) that are important factors in the process of fibrosis. Data concerning MMP expression in equine endometrial fibrosis are limited but MMP-2 and MMP-9 (now called 72 kDa gelatinase and 92 kDa gelatinase, in the horse), seem to be involved in this process (11–14). MMP-2 and MMP-9 are gelatinases that denature collagens (gelatins) and other ECM substrates (15, 16). The endogenous inhibitors of MMPs are tissue inhibitors of metalloproteinases (TIMPs) and neutralize the activity of MMPs. Among the four types of TIMPs, TIMP-1 is a specific inhibitor for MMP-9 (15) while TIMP-2 regulates MMP-2 activity (17). A key feature of fibrosis is the disbalance between MMPs and TIMPs resulting the loss of the homeostasis between fibrolysis and fibrogenesis (18).

Novel findings implicate a role for epigenetic modifications contributing to the progression of fibrosis by alteration of gene expression profiles (19–22). Epigenetic modifications, heritable changes in the genome that do not alter the DNA sequence, influence, or regulate gene expression (23). Epigenetic changes, unlike genetic alterations, can be reversed (24) as thus extremely promising for therapeutic use. In mammals, the most studied epigenetic events are DNA methylation and histone modifications, such as methylation, acetylation, ubiquitination, and phosphorylation (25, 26). DNA methylation constitutes a major epigenetic modification of the genome and is essential for cellular reprogramming, tissue differentiation, and normal development related to many biological processes including gene expression regulation. DNA methylation is known to occur at the 5′ of cytosine in CpG dinucleotides which are found mostly in so-called CpG islands present in promoters (27–29) and is catalyzed by DNA methyltransferases (DNMTs) such as DNMT1, DNMT3a, and DNMT3b (30). These CpG islands are enriched in promotor regions close to transcriptional start sites and their methylation might prevent the transcription of the respective gene (31, 32). Hypermethylation of a promoter has long been well recognized as an efficient means of repressing transcription (33). The majority of CpG sites outside of CpG islands are methylated, suggesting a role in the global maintenance of the genome, while the majority of CpG islands in gene promoters are unmethylated, which allows active gene transcription (34, 35). Transcriptional factors bind to the unmethylated promotor region of a gene to allow its transcription. But, if that region becomes hypermethylated this binding does not occur, and this transcription is not activated. However, most recently, it was demonstrated that intragenic DNA methylation could also affect the gene expression (36). In fact, differential methylation within the gene body plays a role in several gene regulation processes (37). One way to evaluate epigenetics mechanisms is through DNA methylation by DNA methyltransferases (DNMT1, DNMT3A, and DNMT3B). However, this only reflects the level of these enzymes, which in turn may indicate the level of global methylation. Another way to assess the DNA methylation pattern is by bisulfite pyrosequencing. This method is very accurate and is commonly used for quantitative analysis of DNA methylation at single nucleotide level, and in a particular region of the gene (CpG islands), providing more detailed information.

As such, we proposed to evaluate the transcriptomic pattern of some of the most relevant genes involved in mare endometrosis (*COL1A1, COL1A2, COL3A1, MMP2, MMP9, TIMP1*, and *TIMP2*) and secondly, perform epigenetic studies of the genes that have shown alterations (through DNA methylation analysis) to determine whether there is an epigenetic regulation of endometrial fibrosis in mares.

## Materials and methods

### Animals

During the breeding season, Lusitano cyclic mares (*n* = 24; 6 per Kenney and Doig's category; 3 in luteal phase and 3 in follicular phase per category) were used for endometrial biopsy procurement. Mare's internal genitalia were examined by transrectal ultrasonography (Sonovet 600). Endometrial biopsies were randomly obtained from cyclic mares (May to July), with a biopsy alligator jaw forceps (ref. 141965; Kruuse), complying to welfare mandates as a clinical procedure, and with owner's consent. The age of mares ranged from 3 ± 0 years within category I; from 3 to10 years (5.17 ± 1.38) in category IIA, from 6 to 14 years (9.50 ± 1.26) in category IIB, and 11 to 23 years (18.5 ± 2.72) in category III.

The tissue was divided into small pieces with a scalpel and then immersed in RNA later for qPCR or in 4% formaldehyde solution for histopathological evaluation. Formaldehyde-fixed endometrium was paraffin embedded and hematoxylin (05-06014E; Bio-Optica) and eosin (HT1103128; Sigma-Aldrich) stained sections were examined under a light microscope (Leica DM500; Leica Microsystems, Mannheim, Germany). Endometrial biopsies were graded based on the extent of inflammation and /or fibrosis, following Kenney and Doig's classification (38). They were assigned to category I (*n* = 6) when the endometrium was healthy or with slight or sparse inflammation or fibrosis; to category IIA (*n* = 6) when there was mild and scattered inflammation and fibrosis; to category IIB (*n* = 6), when moderate inflammation or fibrosis were present; or to category III (*n* = 6), characterized by severe irreversible fibrosis and/or inflammation.

### Real time PCR

Endometrial biopsies, from different Kenney and Doig's categories, were used for the evaluation of *COL1A1, COL1A2, COL3A1, MMP2, MMP9, TIMP1*, and *TIMP2* transcripts, after RNA isolation, cDNA synthesis and qPCR studies, performed as described (39). Briefly, total RNA was extracted using TRI Reagent (Ref T9424; Sigma Life Science), including a DNA-digestion step with an RNase-free DNase (Ref. 79254, RNase-Free DNase Set, Qiagen, Germany), according to manufacturer's instructions. Quantification and quality of RNA was carried out with a Nanodrop system (ND; Fisher Scientific, Spain) and by agarose gel electrophoresis, respectively. The cDNA was obtained from total RNA (1μg), using M-MLV Reverse transcriptase (Ref. M1705; Promega) and oligo (dT) 15 primer (Ref. C101; Promega). Specific primers were designed, as well as the reference gene (Supplementary Table 1), using the Internet-based program Primer-3 (40) and Primer Premier software (Premier Biosoft Interpairs). Mitochondrial ribosomal protein L32 (*MRPL32*) was chosen as the most stable internal control gene (41) among four validated reference genes, as described (39). Using Power SYBER Green PCR Master Mix (Ref. 4367659; Applied Biosystems) and a StepOne-Plus TM Real-Time PCR System (Applied Biosystems), qPCR studies of target and reference genes were performed simultaneously. Zhao and Fernald (42) method was used to analyze the relative mRNA data.

### DNA preparation

DNA extraction was performed using the kit Quick-DNA 96 Plus Kit (Zymo Research®). Briefly the biopsy sample (25 mg) was diluted with 95 μL of DEPC water and 95 μL Buffer Solid Tissue as indicated in the Kit. The samples were then macerated using the TissueLyser (QIAGEN), 5 cycles of 25 mHz for 30 s. The protocol was performed as instructed by the Kit. Quantification and quality of DNA was carried out with a Nanodrop system (ND; Fisher Scientific, Spain). 500 μL of each sample (24 in total; 6 per Kenney and Doig's category) were sent to an external lab for DNA bisulfite pyrosequencing methylation analysis.

### Promoter annotations

CpG islands and CG percentage were predicted for *COL1A1, MMP2, MMP9* and *TIMP1* equine gene sequences with MethPrimer software (43). Within these, hotspot regions with the highest percentage of CpGs were identified and used in our analysis (Supplementary Table 2). Among the four genes (*COL1A1, MMP2, MMP9*, and *TIMP1*) studied, three were annotated in this study to possess CpG islands within the promoter region (*COL1A1, MMP2* and *TIMP1*). Only *MMP9* did not have CpG islands in the first 1,000 bp and therefore the studied region was exon 8, by homology with human studies, which have demonstrated regulation of *MMP9* gene transcription in this region (44).

### DNA bisulfite pyrosequencing analysis (CpG Islands)

The bisulfite modified DNA sample was then 10-fold diluted and 1 μL of diluted DNA was used in PCR reactions with 3 μL 10xPCR buffer, 200 μL/L of dNTPs, 6 pmol forward primer, 6 pmol reverse primer, and 3 mmol/L MgCl_2_, 0.75 U Qiagen HotStar Taq polymerase (Qiagen Inc., Valencia, CA., 205203) in 30 μL total volume adjusted using double distilled H_2_0, as necessary. The PCR cycling condition was as follows: 95 °C 15 min; 45 x (95 °C 30 s; 51 °C 30 s: 72 °C 30 s); 72°C 10 min; 4 °C∞. The PSQ96HS system was used according to standard procedures for the Pyrosequencing TM analysis. DNA methylation pattern of *COL1A1, MMP2, MMP9*, and *TIMP1* was analyzed by bisulfite pyrosequencing in an external lab (IMIBA, Malaga, Spain). Quantitative sodium bisulfite pyrosequencing was performed, as previously described (45), for the genes that showed differences in the transcriptomic analysis between endometrial categories, and comprised: *COL1A, MMP2, MMP9* and *TIMP1*. In brief, targeted assays were designed using the PyroMark Assay Design Software 1.0 (Qiagen). Forward, reverse, and sequencing primers were used for PCR and pyrosequencing (Supplementary Table 2). The % of methylation was calculated as a mean of the CpG sites that passed quality control. Samples were considered for the study where at least 80% of the CpG sites passed quality control.

### *In silico* analysis

The *in-silico* analysis was performed in the genes where methylation was correlated to transcription: *MMP2* and *MMP9* genes. CpG islands sequences were analyzed using two different programs: TRANSFAC® database (46) and Alibaba 2.1 (47) in search for possible binding transcription factors of the regulatory region of the genes (Figure 6).

### Statistical analysis

Normal distribution of the data was evaluated by Shapiro-Wilk test. Kruskal-Wallis analysis followed by Dunn's multiple comparison test were performed to compare *COL1A1* and *MMP9* transcripts and *COL1A1, TIMP1*, and *TIMP2* methylation between endometrial categories. One-way analysis of variance (ANOVA) followed by *post-hoc* Tukey multiple comparison test were used to analyse *COL1A2, COL3A1* and *TIMP2* mRNA and *MMP9* methylation, between endometrial categories. Unpaired *t*-test was used to compare *MMP2* and *TIMP1* mRNA and *MMP2* methylation, between endometrial categories. Pearson correlation test was performed to analyse transcription and methylation of *MMP2* and *MMP9* genes. GraphPAD PRISM (Version 8.1.0, 253, San Diego, CA, USA) was used. Significance was considered when *P* < 0.05. Data are presented as mean ± SEM.

## Results

### *MMP2* and *MMP9* expression is downregulated during fibrosis progression

**Figure 1:**
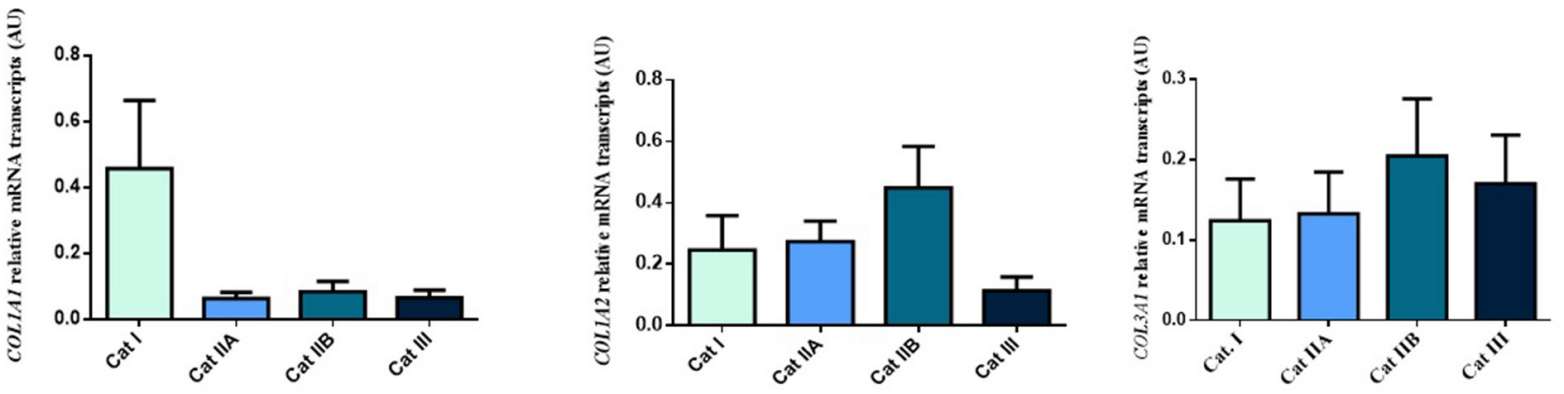
Relative *COL1A1, COL1A2* and *COL3A1* mRNA transcripts in equine endometrium graded as Kenney and Doig's categories I, IIA, IIB and III. Bars represent mean ± SEM.

**Figure 2:**
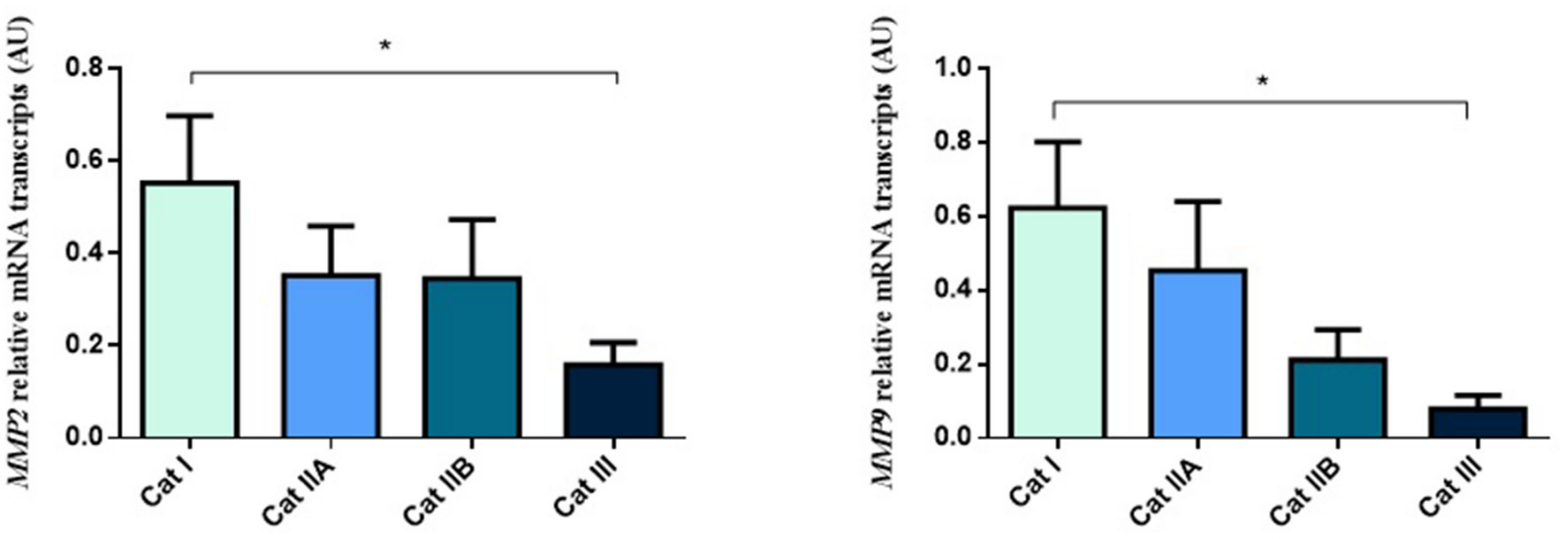
Relative *MMP2* and *MMP9* transcripts in equine endometrium graded as Kenney and Doig's categories I, IIA, IIB and III. Bars represent mean ± SEM. The asterisk indicates significant differences between endometrial categories (*P < 0.05).

**Figure 3:**
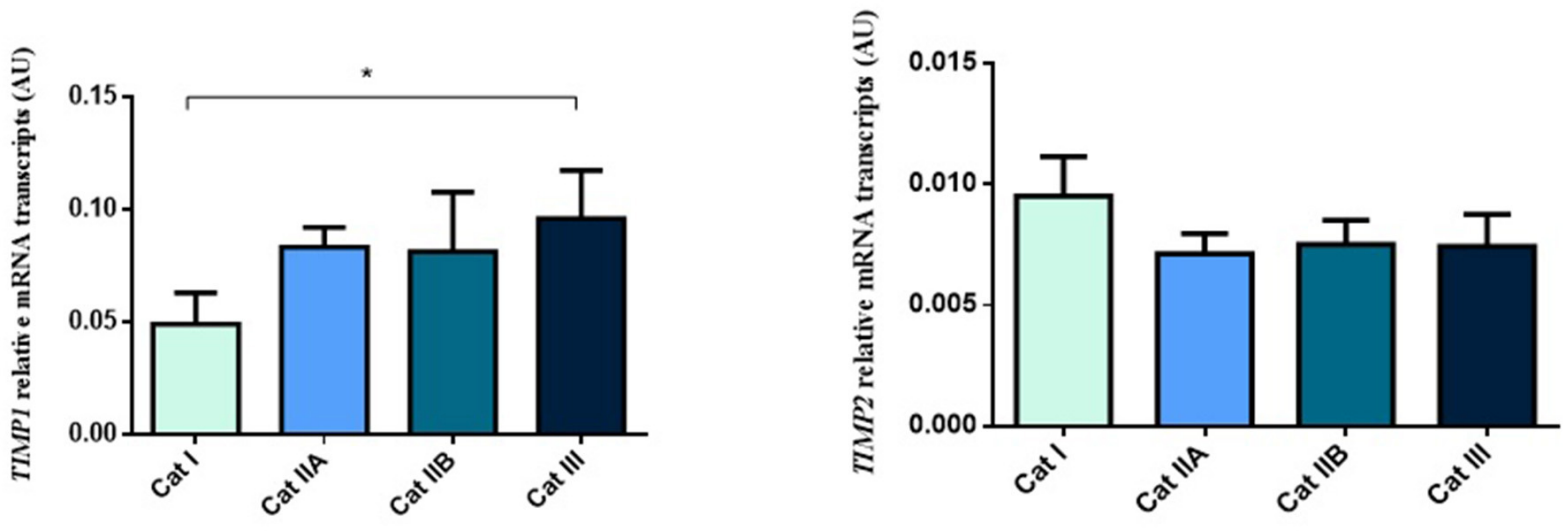
Relative *TIMP1* and *TIMP2* transcripts in equine endometrium graded as Kenney and Doig's categories I, IIA, IIB and III. Bars represent mean ± SEM. The asterisk indicates significant differences between endometrial categories (*P < 0.05).

Firstly, we interrogated if expression levels of the COL genes and some of their regulators (MMPs and TIMPs) were altered in the different endometrial categories. Although no statistical differences in mRNA levels were observed in *COL1A1, COL1A2* and *COL3A1* between different stages of endometrosis (Figure 1), a striking decrease in mRNA levels was observed for the *MMP2* and *MMP9* metallopeptidase genes (*P* < 0.05) between endometrial categories (Figure 2). As for the metallopeptidase tissue inhibitors studied, *TIMP1* transcripts were higher in category III when compared to category I endometrium (*P* < 0.05) whereas no differences were found for *TIMP2* transcripts (Figure 3). Mares were then grouped by phase of estrous cycle for each category and no differences were found between luteal and follicular phase (data not shown).

### DNA methylation plays a role in endometrial fibrosis regulation

**Figure 4:**
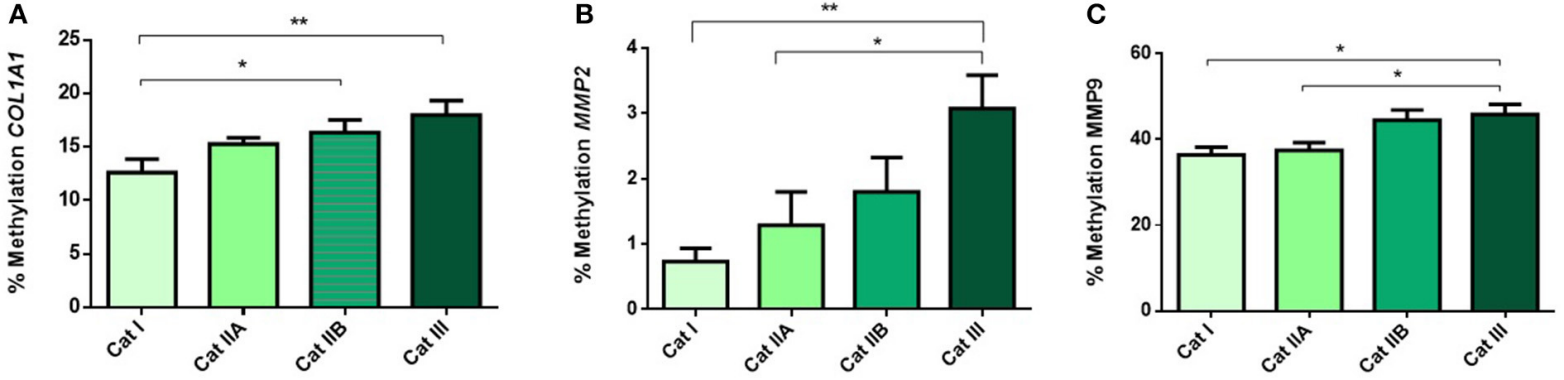
Methylation of DNA (%) of **(A)** *COL1A1*, **(B)** *MMP2* and **(C)** *MMP9* in equine endometrium graded as Kenney and Doig's categories I, IIA, IIB and III. Bars represent mean±SEM. The asterisks indicate significant differences between endometrial categories (*P < 0.05, **P < 0.01).

Next, we questioned if the observed alterations in mRNA levels of the studied genes were associated with epigenetic mechanisms. As such, we performed DNA methylation analysis and observed higher methylation levels of *COL1A1* in category IIB (*P* < 0.05) and III (*P* < 0.01), when compared to category I endometrium (Figure 4A). In addition, higher levels of methylation were observed for *MMP2* in category III when compared to category I (*P* < 0.001) and IIA (*P* < 0.05) (Figure 4B) and for *MMP9* gene in category III with respect to category I and IIA endometrium (*P* < 0.05) (Figure 4C). No difference in methylation levels between endometrium categories were observed for *TIMP1* (data not shown). There were no methylation differences between luteal and follicular phase for each endometrial category.

### Methylation of *MMP2* and *MMP9* is negatively correlated with its transcription

**Figure 5:**
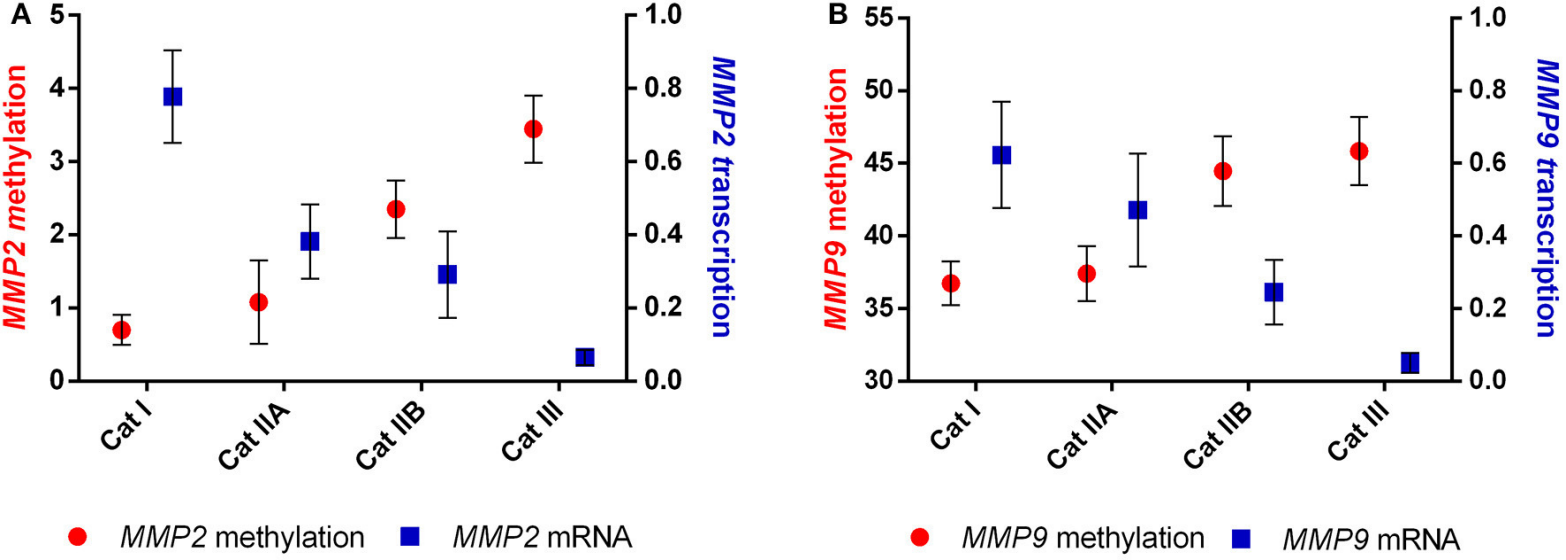
Correlation between methylation and transcription of **(A)** *MMP2* and **(B)** *MMP9* in equine endometrial categories graded as Kenney and Doig's classification.

We than analyzed the correlation between the observed DNA methylation events and potential alterations in gene expression. The higher methylation levels observed in *MMP2* and *MMP9* were strongly correlated with the decreased transcription levels of both genes (*r* = −0.967, *P* < 0.05 and *r* = −0.956, *P* < 0.05, respectively) upon endometrial fibrosis progression (Figure 5).

### Sp-1 and Ap-2α transcription factors identified as predicted binding factors in regulatory regions of *MMP2* and *MMP9*

**Figure 6:**
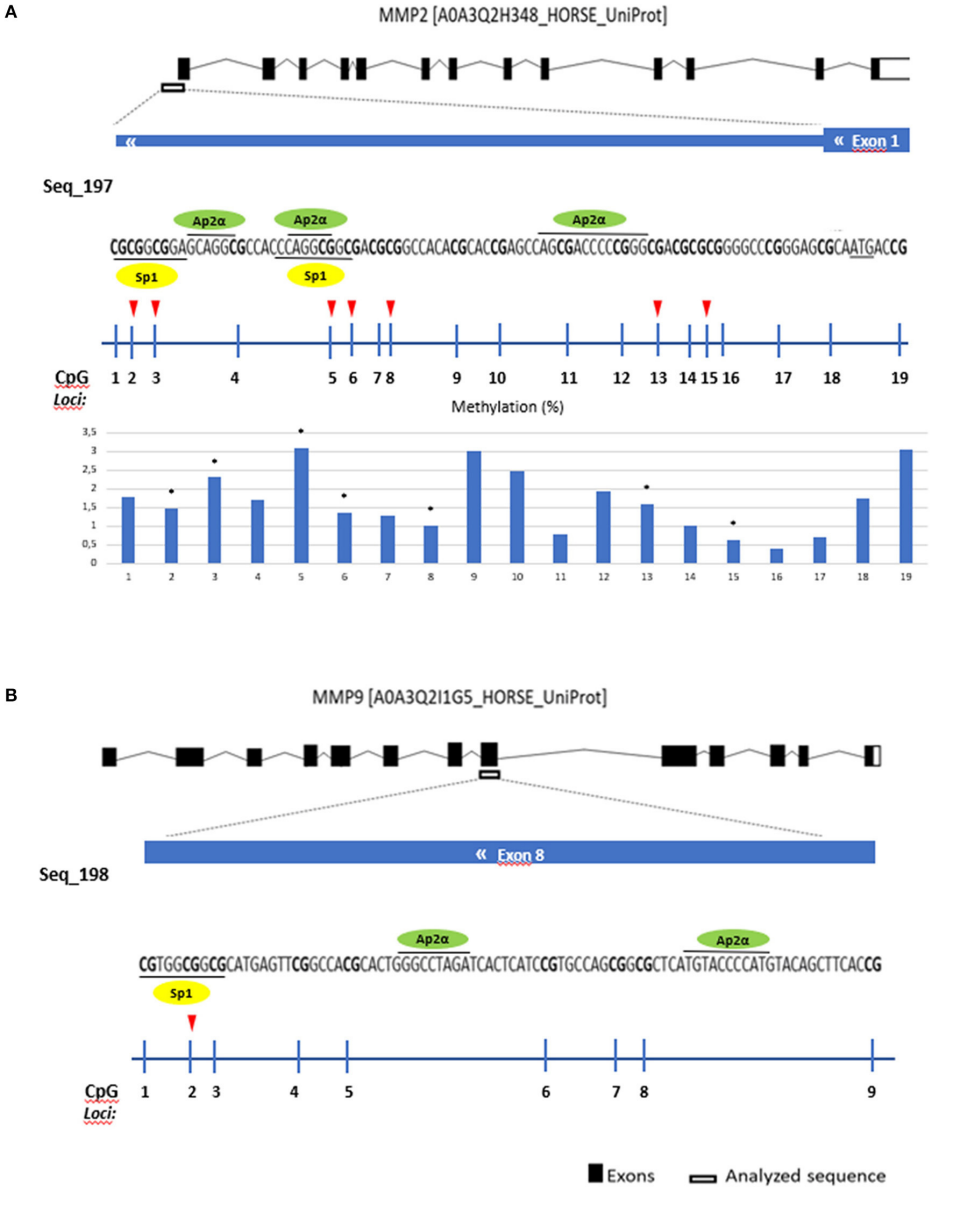
Gene analyzed sequence, binding transcription factors and methylation (%) in equine endometrial categories graded as Kenney and Doig's classification per CpG position in **(A)** *MMP2* and **(B)** *MMP9*. Sp1 - proximal specificity protein 1, AP2α- Activator protein 2. Red arrows and asterisks indicate significant differences between CpG positions (*P < 0.05).

Several transcription factors from each software program were detected and the overlapping factors from the two programs were identified. The predicted transcription factors for *MMP2* and *MMP9* were Sp-1 and Ap-2α (Figure 6).

**Figure 7:**
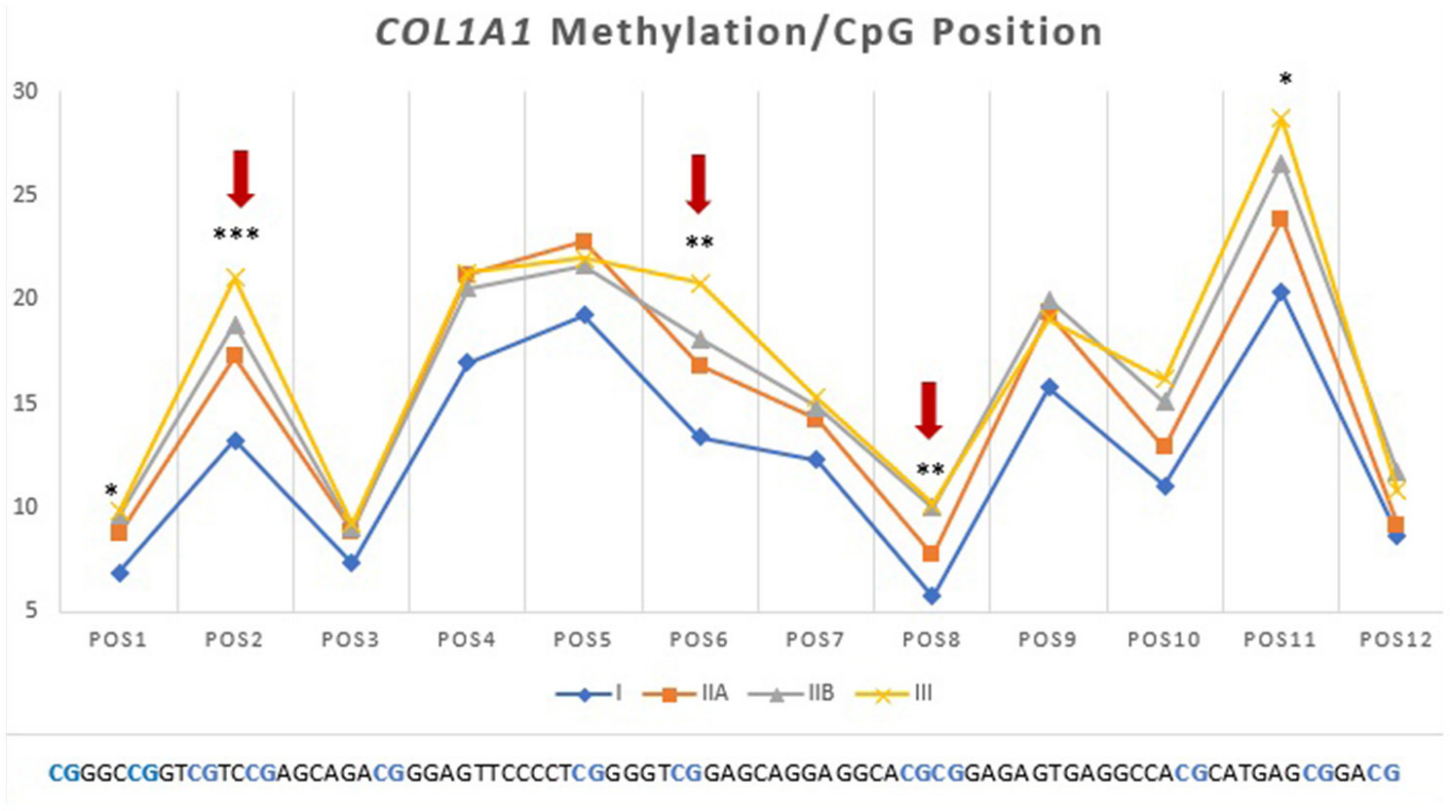
Methylation (%) of *COL1A1* by CpG position in equine endometrial categories graded as Kenney and Doig's classification. Red arrows and asterisks indicate significant differences between CpG positions (*P < 0.05, **P < 0.01, ***P < 0.001).

Then we analyzed which positions within the CpG island were accountable for the observed alterations. For *COL1A1* the CpG positions that showed more alterations in methylation levels between endometrial categories were sites 2, 6, and 8 (Figure 7); for *MMP2* site 2, 3, 5, 6, 8, 13, and 15 (Figure 6A); and *MMP9* only site 2 (Figure 7B).

## Discussion

Novel findings implicate a role for epigenetic modifications contributing to the progression of fibrosis by alteration of gene expression profiles. Furthermore, accumulating evidence suggests that epigenetic alterations are central in maintaining the myofibroblast phenotype (20). Several studies demonstrated that the hypermethylation of gene promotors of antifibrotic mediators plays important roles in pathologic fibroblast activation and that inhibition of DNMTs prevents fibrosis in many fibrotic diseases (48–55). Previously, we reported an increase in *DNMT3B* mRNA levels with equine endometrial fibrosis (category III) when compared with healthy endometrium (category I), indicating there was hypermethylation with the advance stage of endometrosis (56). However, more detailed information is needed to understand which genes were involved and if hypermethylation was occurring in the gene promoter region and therefore could be regulating its transcription.

Our first proposed goal to evaluate transcriptomic of some of the genes involved in mare endometrial fibrosis, showed a decrease in *MMP2* and *MMP9* mRNA transcripts and an increase in *TIMP1* mRNA transcripts with endometrial fibrosis. Although the *COL1A1* mRNA transcripts did not show statistical differences we decided to include it in the DNA methylation study, along with the genes where an alteration was observed, to understand if they were epigenetically regulated.

The main constituents of fibrotic lesions are interstitial collagens, such as COL1 and COL3, and excessive deposition of these durable fibers can result in disruption of proper tissue structure and function (17). Activated fibroblasts are the central mediators in the pathogenesis of fibrosis and they differentiate into myofibroblasts, which are characterized by the increased secretion of collagen and other components of the extracellular matrix (57, 58). In the present study, no differences were found for *COL1A1, COL1A2* or *COL3A1* transcripts between different endometrial categories. In a study in equine endometrium (59) neither the estrous cycle phase, nor the age of mares had any effect on *COL1* mRNA levels. Another study in jennies showed no differences in *COL1A2* and *COL3A1* transcripts between endometrial categories (60). However, some *in vitro* studies with mare cultured endometrial fibroblasts showed increased *COL1A1* and *COL3A1* mRNA transcripts after TGF-β1induced fibrosis (61) together with high COL1 and COL3 protein levels. Additionally, a study in equine tendon fibroblasts also demonstrated an increase in *COL1A1* and *COL1A2* gene expression after stimulation with TGF-β (62). This divergence could be due to differences in signaling and regulatory pathways in tissues examined *ex vivo* or cultured *in vitro*. Nevertheless, data from other study, with the same mares (unpublished data), have shown elevated COL1 and COL3 protein concentrations in endometrial tissue with fibrosis, regardless of unaltered *COL1* and *COL3* gene transcription. This suggests that accumulation of collagen in the endometrial tissue might be due to lack of degradation, rather than increased collagen production. Other explanation could be that the mRNA signal for COL1 and COL3 production might have occurred before the biopsy was performed. It is known that mRNAs are less stable than proteins with a maximum half-life of approximately 7 h, compared to 46 h for proteins (63). Thus, protein abundance mainly depends on a dynamic balance amongst transcription, mRNA processing and damage, translation, modification, and destruction of the resulting proteins (63). DNA methylation of the CpG islands in the promotor zone of the *COL1A1* gene increased with endometrial fibrosis. However, hypermethylation of *COL1A1* gene with fibrosis, did not result in its altered transcription. It is important to consider that collagen synthesis is precisely controlled at multiple levels, including *via* post-transcriptional and post-translational mechanisms that are still being discovered (64).

One class of molecules that is thought to be important in the maintenance of the ECM and processes of tissue repair is the class of matrix metalloproteinases (MMPs). The MMPs have been considered to play an important role in the extracellular matrix turnover (15) and a balance between activation and inhibition of MMPs is crucial for maintaining tissue homeostasis (14). Dysregulated expression of various MMPs is associated with many pathological processes, such as fibrosis, weakening of ECM or tissue destruction, e.g., in cancer metastasis (65, 66). However, data concerning MMP expression in equine endometrium during endometrial fibrosis is limited. In our study both *MMP2* and *MMP9* gene expression decreased with endometrial fibrosis, suggesting that the reduced transcription may result in diminished degradation of collagen and its accumulation in the endometrium. Other studies also demonstrated lower *MMP2* gene transcription in fibrotic endometrium but *MMP9* transcription was higher (67). In another study with endometrial fibroblasts in mares (14), *MMP9* gene transcription increased after TGF-β1 stimulation. Also, a study by Centeno and collaborators (12) showed that *MMP2* transcription was upregulated in endometrial fibrosis. On the other hand, a study in mice with induced liver fibrosis reported increased *MMP2* mRNA and decreased *MMP9* mRNA (68). Many studies in other animals and humans have shown a decrease in *MMP2* (69, 70) and *MMP9* gene expression in several fibrotic diseases (71) while others reported the opposite (72–74). These inconsistent results regarding *MMP2* and *MMP9* expression might be partially explained by the fact that MMPs demonstrate tissue-dependent and disease-specific expression and function (75). Moreover, current knowledge about MMP regulation is largely based on cell culture systems, raising a major question as to whether identical mechanisms apply to MMP expression in the whole organism as well (76).

**Figure 8:**
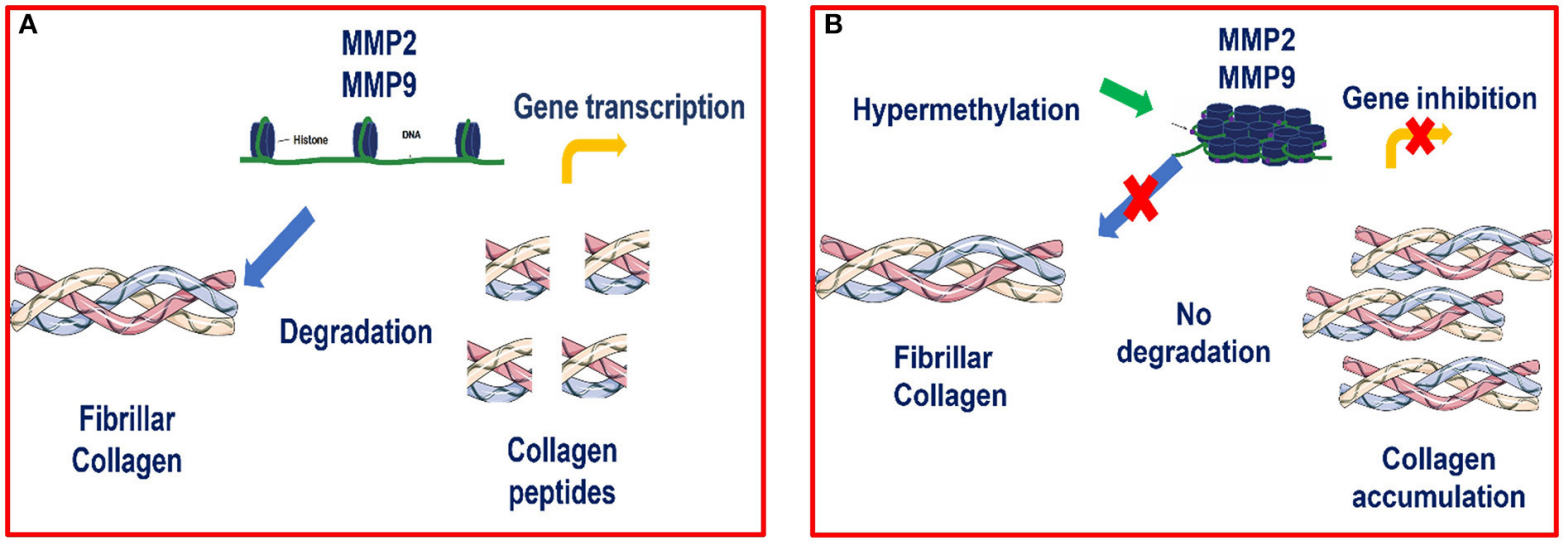
**(A)** Transcription of *MMP2* and *MMP9* genes is possible when there is no hypermethylation of these genes, as chromatin is accessible and thus allows transcriptional factors to bind to the promoter region and activate the transcription process **(B)** Epigenetic modulation of *MMP2* and *MMP9* in equine endometrial fibrosis. When hypermethylation occurs, the chromatin becomes condensed and the transcriptional factors are not able to bind to this site and therefore, transcription is inhibited.

Our results showed increased methylation in *MMP2* and *MMP9* in category III when compared to category I and IIA. Our study agrees with many others in that hypermethylation of antifibrotic gene promotors is involved in fibrosis development (48–54). Furthermore, we observed that hypermethylation of *MMP2* and *MMP9* genes occurred concomitantly with a decrease in their transcription levels as fibrosis increased, showing an epigenetic regulation as suggested in Figure 8. Other studies also observed that silencing of MMP *genes* is likely mediated by epigenetic alterations (68).

Tissue inhibitors of metalloproteinases (TIMPs) are the major endogenous regulators of MMP activities in the tissue microenvironment and have the capacity to modify cellular activities and to modulate matrix turnover (77). TIMP-1 and TIMP-2 proteins bind to and inhibit activated collagenases, subsequently protecting newly synthesized collagen from immediate degradation by MMPs (18). In our study *TIMP1* mRNA transcripts raised with fibrosis when compared to healthy endometrium whereas no differences were observed for *TIMP2* mRNA. Our data agrees with a study in human lung fibrosis, where *TIMP1* mRNA was markedly increased in response to lung injury, whereas there was no change in *TIMP2* mRNA levels (77). Also, studies by Heymans in human cardiac disease (78) and Wang in rat lungs (79) reported upregulated TIMP1 gene expression during fibrosis. Since in our study, there were no alterations in DNA methylation pattern between endometrial categories for the TIMP1 gene, this might suggest that its inhibition does not occur, continuing to be expressed and increased with fibrosis, thus contributing to collagen accumulation in mare endometrium.

Some of the studied CpG island positions showed more differences in methylation between categories than others. Transcriptional factors (TF) play an important role in gene transcription as they can regulate transcription by binding to the activator or promoter regions of DNA and control gene expression through various mechanisms (80). The *in silico* analysis performed (81, 82) in the analyzed regions of the *MMP2* and *MMP9* genes, detected Sp-1 and Ap-2α as possible binding transcription factors for *MMP2* and *MMP9*. These putative binding regions encompass CpG sites that showed significant methylation differences across the four studied endometrial categories. Specificity protein 1 (Sp-1) binding sites are often located close to binding sites for activator proteins (Ap-1/Ap-2) (83), as it was observed in our analysis. Transcription factor Ap-2, an important TF for the expression of many genes (84) has a role in the transcriptional regulation of *MMP2* in humans (85, 86). In this study, the binding region for Sp-1 in *MMP9* comprised loci 2, where the differences in methylation between endometrial categories occurred. Therefore, we speculate that these factors may play a role in the complex mechanism of endometrial fibrosis regulation and should be further investigated.

Overall, it seems like a different approach might be needed to address fibrosis treatment, as up to date, no effective therapy exists for equine endometrosis. “Epi-drugs” that target active myofibroblasts in fibrotic disorders are a promising direction in the treatment of a myriad of diseases. Nevertheless, we are far from a comprehensive understanding of how epigenetic modulators influence each other and myofibroblast behavior (23). If epigenetic mechanisms are involved in mare endometrial fibrosis development, as suggested by our results, then therapeutic agents that can reverse these epigenetic changes may represent a new and promising approach, for a condition that still has no available treatment.

## Conclusion

In this study we have showed that equine endometrial fibrosis seems to be epigenetically modulated. Furthermore, that modulation seems to occur through the inhibition of antifibrotic genes like *MMP2* and *MMP9*, rather than fibrotic genes (*COL1* and *TIMP1*) and therefore might be promising targets for therapeutic use. Nevertheless, further studies are required to understand in depth this mechanism and possible role of other genes involved in mare endometrosis.

## Data availability statement

The original contributions presented in the study are included in the article/Supplementary material, further inquiries can be directed to the corresponding author.

## Ethics statement

The animal study was reviewed and approved by Ethic Committee for Research and Teaching Comissão de Ética para a Investigação e Ensino-CEIE (Ethic Committee No: 013/2022), Faculty of Veterinary Medicine, University of Lisbon, Lisbon, Portugal. Written informed consent was obtained from the owners for the participation of their animals in this study.

## Author contributions

Conceptualization: JA-M, GF-D, MR, and DS. Methodology: JA-M, CF, and MB. Software and writing—original draft preparation: JA-M. Validation: GF-D, JP, PC-B, and DS. Formal analysis: JA-M and CF. Investigation: JA-M, JP, and MR. Resources: JP and MB. Data curation: JA-M, JP, and MR. Writing - review and editing: JA-M, JP, PC-B, MR, AS, and GF-D. Visualization and funding acquisition: GF-D and DS. Supervision: GF-D and MR. Project administration: GF-D. All authors contributed to the article and approved the submitted version.

## Funding

UIDB/00276/2020; PTDC/CVT-REP/4202/2014; LA/0059/2020/ AL4AnimalS funded by Fundação para a Ciência e Tecnologia (FCT; Portugal), OPUS19 nr 2020/37/B/NZ9/03355 funded by the National Science Center, (NSC; Poland), and bilateral Polish-Portugal project under NAWA and FCT agreement (2019–2020).

## Conflict of interest

The authors declare that the research was conducted in the absence of any commercial or financial relationships that could be construed as a potential conflict of interest.

## Publisher's note

All claims expressed in this article are solely those of the authors and do not necessarily represent those of their affiliated organizations, or those of the publisher, the editors and the reviewers. Any product that may be evaluated in this article, or claim that may be made by its manufacturer, is not guaranteed or endorsed by the publisher.
