# Supplementary material for: Metallopeptidades 2 and 9 genes epigenetically modulate equine endometrial fibrosis
Source: Front Vet Sci. 2022 Aug 12;9:970003. doi: 10.3389/fvets.2022.970003 (PMC9412240; doi:10.3389/fvets.2022.970003)
Supplement: Supplementary file 1 [file Data_Sheet_1.docx]

Supplementary Material

# Supplementary Tables

## Supplementary Table 1

Supplementary Table 1 – Primer sequences used in qPCR study

| **Gene**  **(Acession number)** | Sequence 5´- 3 | | **Amplicon**  **(base pairs)** |
| --- | --- | --- | --- |
|  | **Forward** | **Reverse** |  |
| *COL1A1*  (XM_023652710.1) | TATGGAAACCCGAGCCCTG | ACTCCTGTGGTTTGGTCGTCTG | 175 |
| *COL1A2*  (XM_001492939.3) | CAAGGGCATTAGGGGACACA | ACCCACACTTCCATCGCTTC | **196** |
| *COL3A1*  (AF117954.1) | CAAAGGAGAGCCAGGAGCAC | CTCCAGGCGAACCATCTTTG | **98** |
| *MMP2*  (XM_001493281.2) | TCCCACTTTGATGACGACGA | TTGCCGTTGAAGAGGAAAGG | **115** |
| *MMP9*  (NM_001111302.1) | GCGGTAAGGTGCTGCTGTTC | GAAGCGGTCCTGGGAGAAGT | **177** |
| *TIMP1*  (NM_001082515.1) | CAAGTTCGTGGGGACCTCAG | CTCTCCATAGCGGGGGTGTA | **141** |
| *TIMP2*  (XM_023651899.1) | ATCTACGGCAACCCCATCAA | CTTCTTCCCTCCAACGTCCA | 144 |
| *RPL32*  (XM_001492042.6) | AGCCATCTACTCGGCGTCA | GTCAATGCCTCTGGGTTTCC | 144 |

*COL1A1* - collagen type 1, alpha1; *COL1A2* - collagen type 1, alpha2; *COL3A1* - collagen type 3, alpha1; *MMP2* - matrix metalloproteinase 2; *MMP9 -* matrix metalloproteinase 9; *TIMP1* - tissue inhibitor of matrix metalloproteinase 1; *TIMP2* - tissue inhibitor of matrix metalloproteinase 2; *RPL32* - ribosomal protein L32

## Supplementary Table 2

Supplementary Table 2 - Primer sequences used in DNA methylation study.

| **Gene** | **PCR Primers** | | **Target region (length)** | **CpG sites (nº)** |
| --- | --- | --- | --- | --- |
|  | **Forward** | **Reverse** |  |  |
| *COL1A1* | GGGTAGGGTTAGGTAGTTTTGATT | CATATCTAAACCCTAAACATATAAACTCTT | 85 bp | 12 |
| *MMP2* | GGGGTTTTAAATATATAAAGGGATTGT | ACATCTCCAAAAAACTTAATAATAAAC | 95 bp | 19 |
| *MMP9* | GGTTGGGAGTTTAGTTTAGGG | CCCACAACCTCACCATAAAAATACT | 61 bp | 8 |
| *TIMP1* | TTTAGGGGGAGGGAGTGG | CCCCCCCTACCTCTACTAAAATCTCTCTA | 103bp | 7 |

*COL1A1* - collagen type 1, alpha1; *MMP2* - matrix metalloproteinase 2; *MMP9 -* matrix metalloproteinase 9; *TIMP1*- tissue inhibitor of matrix metalloproteinase 1
